# Applying a weed risk assessment approach to GM crops

**DOI:** 10.1007/s11248-013-9745-0

**Published:** 2013-09-18

**Authors:** Paul K. Keese, Andrea V. Robold, Ruth C. Myers, Sarah Weisman, Joe Smith

**Affiliations:** Office of the Gene Technology Regulator, GPO Box 9848, Canberra, ACT 2601 Australia

**Keywords:** Environmental risk assessment, GMO regulation, Weed risk assessment, Invasiveness, GM plant, Biosafety

## Abstract

Current approaches to environmental risk assessment of genetically modified (GM) plants are modelled on chemical risk assessment methods, which have a strong focus on toxicity. There are additional types of harms posed by plants that have been extensively studied by weed scientists and incorporated into weed risk assessment methods. Weed risk assessment uses robust, validated methods that are widely applied to regulatory decision-making about potentially problematic plants. They are designed to encompass a broad variety of plant forms and traits in different environments, and can provide reliable conclusions even with limited data. The knowledge and experience that underpin weed risk assessment can be harnessed for environmental risk assessment of GM plants. A case study illustrates the application of the Australian post-border weed risk assessment approach to a representative GM plant. This approach is a valuable tool to identify potential risks from GM plants.

## Introduction

Risk assessment forms the foundation for regulatory decisions on whether to authorize the environmental release of a genetically modified organism (GMO). Risk assessment is a structured, reasoned approach to identify a GMO’s potential to cause adverse effects (harm) and to characterize the seriousness and likelihood of potential harm.

To date, the majority of GMOs approved for environmental release are crop plants. The greatest repository of knowledge and experience of plants (including crops) that cause adverse effects is in the field of weed science. Weed scientists have developed and refined robust weed risk assessment methods (Pheloung 2001; Standards Australia 2006) that are commonly used in decision-making. We suggest that a weed risk assessment approach can be usefully applied in the risk assessment of GM crops.

This paper is based upon presentations at the 12th International Symposium on the Biosafety of Genetically Modified Organisms (ISBGMO 12) held in September 2012 in St. Louis, Missouri.

## Principles of risk assessment

Risk assessment of GMOs feeds into regulatory decision making. Requirements for risk assessment are set out in national biosafety legislation and in international agreements. For example, the United Nations Cartagena Protocol for Biosafety describes risk assessment requirements for the safe transboundary movement of GMOs that apply to parties to the Protocol. In addition, the Organisation for Economic Co-operation and Development produces consensus guidance documents related to risk assessment of GMOs.

Typically, risk assessment includes four key components.

1. Establishing the risk context (planning/scoping) to define what should be considered in the risk assessment and how it should be considered. This includes national and international legal requirements, as well as protection goals that define what is considered harm.
2. Risk identification to postulate scenarios (risk hypotheses/conceptual models) which describe a plausible causal pathway from a source of potential harm (GM trait) to potential harm to an object of value (people or environment).
3. Risk characterization to consider the seriousness (consequences) and likelihood of potential harm for each identified scenario.
4. Risk evaluation to judge the overall significance of risk.

Where possible, a comparative risk assessment approach is used, such that risk from a GMO is considered relative to the parent organism within the environment where the GMO is proposed to be released or may spread. The focus of the assessment is whether traits modified by gene technology increase the level of risk, or give rise to additional risks.

## Relevance of weed risk assessment for GM plants

### GMOs are organisms not chemicals

Currently, many risk assessments of GMOs are based on the framework and terminology established by the US National Research Council in 1983 and revised in 2009 (National Research Council 1983, 2009), which supported the development of an evidence-based regulatory system for GM crops. This framework was based on chemical risk assessment and modified for application to risk assessment of biological organisms (U.S. EPA and USDA/FSIS 2012). However, there are significant differences between the properties of chemicals and organisms that affect the risk assessment (Ahl et al. 2003; U.S. EPA and USDA/FSIS 2012). For example, unlike chemicals, organisms can reproduce and multiply. While chemical risk assessment primarily considers the harm of toxicity, some organisms have a long history of causing harms other than toxicity (e.g. smallpox, locusts, rats, kudzu vines or cape broom). In addition, risk assessments of plants and animals usually consider the possibility of increased invasiveness. Therefore, risk assessment approaches developed specifically for organisms such as pathogens, pests and weeds are highly relevant to GMOs.

### GM phenotypic traits are present in non-GM plants

Genetic modification acts at the first instance at the molecular level, i.e. changes to genes, proteins or metabolites. However, it is the phenotypic trait induced by these changes (e.g. toxicity, herbicide tolerance or abiotic stress tolerance) that potentially impacts on human health or the environment, and therefore is central to the risk assessment. Although an introduced trait in a GM plant may be a novel in that particular cultivar or species, the trait is expected to be present in other cultivars or species, often to a greater extent. For example, many plants have natural toxicity to a broad range of insects, meaning that the phenotypic trait of insect resistance is shared by a broad subset of plants, and is not unique to Bt crops. Similarly, many pharmaceutical compounds are originally derived from plants, so the phenotypic trait of producing compounds with physiological effects on humans is common among plants rather than being restricted to GMOs for biomedical applications. Weed risk assessments are designed to deal with a great variety of plant species and their phenotypic traits, which would encompass consideration of any potential traits of GM plants.

### Weed risk assessment protocols are mature

Distinguishing features of weeds were first formalized by Baker (1965). These have been developed and expanded into modern weed risk assessment protocols. Australian weed scientists have particularly broad experience with weeds, as Australia hosts more than 1,100 major agricultural and environmental weeds (Randall 2012) which invade the wide variety of agroecological zones across the Australian continent. Australian weed scientists have led development of two weed risk assessment approaches: a pre-border screen for proposed novel plant introductions and a post-border approach used to assess plants already present in the environment for purposes of weed management prioritization (Auld 2012). The systems are routinely used for regulatory decision-making, and are continually reviewed and refined in light of experience (Auld 2012).

### Weed risk assessment protocols are validated

Using reliable risk assessment methodology is important for scientific credibility of the predicted level of risk, justification for requiring possible risk management measures and confidence in regulatory decision-making. The reliability of a risk assessment method can only be determined by statistical validation, derived from actual experience of comparing outcomes with predictions (Caley and Kuhnert 2006). It is difficult to validate current risk assessment techniques for GM plants due to the small number of GM plants that have been assessed for environmental release. In addition, there is no unambiguous evidence of approved GM plants causing marked harm to people or the environment, to allow determination of whether decisions to approve or reject environmental release were well founded. In contrast, the large datasets available from weed risk assessments include plants across the whole risk spectrum, and allow rigorous validation tests to be conducted (Stone and Byrne 2011; Virtue et al. 2008).

Table 1 illustrates the success rate of the Australian pre-border weed risk assessment applied to test known invasive and non-invasive plant species across a number of countries (adapted from Gordon et al. 2008). The system was found to correctly reject introduction of almost all major invasive species (99 %) and correctly accept most other test species (90 %). Most errors in the system involve predicting high weed risk for plants that are actually low risk, indicating a cautionary bias.

**Table 1:** Likelihood of correct regulatory decision to approve or reject introduction of test plant species countries (adapted from Gordon et al. 2008)

Status of test species	Number of species approved	Number of species rejected	% of correct decisions
Major invasive species	4	326	99
Non-invasive species	382	41	90

Data is pooled from separate weed risk assessment tests in Australia, New Zealand, Hawaii and Pacific Islands, the Czech Republic, the Bonin Islands of Japan and Florida. Regulatory decisions to require further information are excluded

As weed risk assessment methods have been robustly validated, incorporating them into risk assessment of GM plants can increase scientific rigour.

## The post-border weed risk assessment system

For the purposes of this discussion, the term weed refers to invasive plants that cause harm to the health of people or the environment. Invasiveness refers to a high ability to spread (disperse, expand population) and persist (establish, survive and reproduce). Invasive plants may or may not cause harm (Richardson et al. 2000). In contrast, a plant’s weed status considers both potential invasiveness and adverse impacts.

The Australian post-border weed risk assessment (PBWRA) system is based on scoring answers to a list of questions related to either harm or invasiveness of a plant (Standards Australia 2006). The questions address all plant characteristics known to contribute to weed risk. A combination of seriousness of harm and degree of invasiveness is used to calculate the plant’s comparative weed risk. This indicates which plant species should be considered for weed management. The PBWRA incorporates the Australian/New Zealand Risk Management Standard (Standards Australia 2004). It has been adopted by the Food and Agriculture Organization of the United Nations (2011) and various Australian government departments, agencies and research bodies, including the Australian Gene Technology Regulator (OGTR 2011), with minor modifications to suit their respective regulatory purposes.

The PBWRA is answered separately for each relevant land use (receiving environment). The management objective of a land use may be primary production (e.g. agriculture, forestry), conservation (e.g. nature reserve) or human services (e.g. residential, water supply, roadsides). These types of environments may have varying susceptibility to invasion and different protection goals.

### Application of PBWRA to GM plants

The PBWRA is a valuable tool in risk assessment of GM plants conducted by the Gene Technology Regulator in Australia. Although the PBWRA does not encompass the entire risk assessment process as described in the *Principles of Risk Assessment* section above, it contributes to the first two key steps of risk assessment, establishing the risk assessment context and risk identification.

When establishing the risk assessment context, the PBWRA can provide guidance on the crucial question of what is considered harm. A set of hundreds of known weeds was used in the development of the PBWRA, and a compilation of the harms caused by these weeds covers effectively the entire range of harms that might be caused by plants. It indicates the potential harms that should be considered when assessing GM plants.

Another important component of establishing risk assessment context is to consider the receiving environment, which is determined by those locations where the GMO is predicted to be present, either through deliberate release of plant propagules or through spread of GM plants. The functions of the receiving environment (e.g. nature reserve land use) have a close relationship with the harms that are relevant for risk assessment (e.g. loss of biodiversity). The PBWRA makes a clear distinction between different categories of land use, and facilitates conducting separate assessments for each land use.

Risk assessment of a GM plant generally does not evaluate the plant in isolation, but compares it to a parent non-GM plant, and attempts to determine whether the GM plant poses greater risks to people or the environment than the parent. It is possible to conduct side-by-side PBWRA for the GM plant and its parent in each relevant land use. If an impact or invasiveness characteristic changes significantly due to the genetic modification, this characteristic should be highlighted for further consideration. When postulating risk scenarios for the GM plant, the characteristic imparting increased potential for invasiveness or impact should be taken into account (see case study below).

### Harms from plants

In chemical risk assessments consequences are quantified as dose response, while the consequences of infection with microorganisms are described as virulence. In weed risk assessment, the equivalent term is impact. Adverse impacts, also termed adverse consequences or harms, from plants include death, injury or impairment of desirable organisms. They also include undesirable changes to the quality of the physical environment (e.g. soil, water, air, or climate) or the function of the land use (e.g. agricultural production or nature conservation).

Attribution of harm may not be straightforward, as different values may conflict. For example, a woody plant growing in an agricultural field may have adverse impacts due to lower crop yield and reduced access, but may also be considered beneficial by preventing erosion and providing food and shelter for desirable native species. Deciding on whether the woody plant is a weed or not involves value judgement. Whether or not people consider a plant as causing harm can also depend on the land use. For example, a plant producing large amounts of biomass in a pasture may be considered desirable whereas the same plant may be considered harmful (weedy) in a nature conservation area if it displaces native species. Attribution of harm may vary over time. For instance, a crop plant may be desirable when deliberately planted, but undesirable as a volunteer in a following crop.

When formally assessing potential harms from GM plants, the primary protection goals are established by national biosafety legislation. Commonly these are protection of the health of people and protection of the environment. Additional guidance may be provided by:

- international standards (e.g. International Plant Protection Convention on preventing introduction and spread of pests);
- national or state environmental legislation where value judgements have been made (e.g. declarations of species or environments to be protected, or declarations of noxious organisms that require control measures).

Most relevant legislation and standards provide high level guidance on values that should be incorporated into environmental risk assessment. Several agencies have assembled more specific definitions of environmental harm to assist in the development of detailed risk assessments. An example is ‘Generic Assessment Endpoints for Ecological Risk Assessments’ used by the United States Environmental Protection Agency (U.S. EPA and USDA/FSIS 2012). However, this guidance material was primarily designed to assess risks from chemicals rather than organisms.

In contrast, the PBWRA methodology (Standards Australia 2006) provides a systematic compilation of environmental harms, known as impacts that could be caused by plants in different types of land use. This set of harms is based on long experience of weeds in different environments. Through extensive consultation, it has incorporated broadly accepted societal values. It can be adapted to different environmental objects or characteristics of value, e.g. lists of protected species. It can be adapted to specific regulatory objectives such as regulation of GM plants. The impact questions considered in a weed risk assessment are set out below (adapted from Stone et al. 2008; Virtue 2005 for the purpose of this paper).

- **Impact question 1**. Could the plant reduce the establishment of desired plants?

The rating options are:

- **High**—The plant could stop the establishment of more than 50 % of desired plants (e.g. regenerating pasture, sown crops, planted trees, regenerating native vegetation), by preventing germination and/or killing seedlings, for example by denying them access to soil moisture, sunlight or nutrients. If the plant is itself the desired crop in a particular land use, this question will not apply, but may apply for volunteers in the subsequent season.
- **Medium**—The plant could stop the establishment of between 10 and 50 % of desired plants.
- **Low**—The plant would stop the establishment of less than 10 % of desired plants.
- **None**—The plant would not affect the germination and seedling survival of desired plants.
- **Impact question 2**. Could the plant reduce the yield or amount of desired vegetation?

The rating options are:

- **Very high**—The plant could reduce crop, pasture or forestry yield, or the percentage cover of mature native vegetation by over 50 %. This question considers yield loss or suppression of established vegetation; failure to establish is covered by impact question 1.
- **High**—The plant could reduce yield or amount of desired vegetation by between 25 and 50 %.
- **Medium**—The plant could reduce yield or amount of desired vegetation by between 10 and 25 %.
- **Low**—The plant would reduce yield or amount of desired vegetation by up to 10 %.
- **None**—The plant would have no effect on growth of the desired vegetation or the plant may become desirable vegetation at certain times of year (e.g. providing useful summer feed), which balances out its reduction in the growth of other desirable plants.
- **Impact question 3**. Could the plant reduce the quality of products or services obtained from the land use?

The rating options are:

- **High**—For *agriculture*, the plant could severely reduce product quality such that it cannot be sold (e.g. due to severe contamination, toxicity, tainting and/or abnormalities). For *native vegetation*, the plant could severely reduce biodiversity (diversity and abundance of native plants and animals) such that it is not suitable for nature conservation and/or nature-based tourism. For *urban* areas, the plant could cause severe structural damage to physical infrastructure such as buildings, roads, and plumbing.
- **Medium**—For *agriculture*, the plant could substantially reduce product quality such that it is sold at a much lower price for a low grade use. For *native vegetation*, the plant could substantially reduce biodiversity such that it is given lower priority for nature conservation and/or nature-based tourism. For *urban* areas, the plant could cause some structural damage to physical infrastructure.
- **Low**—For *agriculture*, the plant would slightly reduce product quality, lowering its price but still passing as first grade product. For *native vegetation*, the plant would have only marginal effects on biodiversity but is visually obvious and degrades the natural appearance of the landscape. For *urban* areas, the plant would cause negligible structural damage, but reduces the aesthetics of an area through untidy visual appearance and/or unpleasant odour.
- **None**—The plant would not affect the quality of products, services or biodiversity.
- **Impact question 4**. Could the plant restrict the physical movement of people, animals, vehicles, machinery and/or water?

The rating options are:

- **High**—Plant infestations could be impenetrable throughout the year, preventing the physical movement of people, animals, vehicles, machinery and/or water.
- **Medium**—Plant infestations would be rarely impenetrable, but could significantly slow physical movement throughout the year.
- **Low**—Plant infestations would never be impenetrable, but would significantly slow physical movement at certain times of the year or provide a minor obstruction throughout the year.
- **None**—The plant would have no effect on physical movement.
- **Impact question 5**. Could the plant affect the health of animals and/or people?

The rating options are:

- **High**—The plant could be highly toxic and frequently causes death and/or severe illness in people, stock, and/or other desirable organisms.
- **Medium**—The plant could occasionally cause significant physical injuries (due to spines or barbs) and/or significant illness (chronic poisoning, strong allergies) in people, stock, and/or other desirable organisms, occasionally resulting in death.
- **Low**—The plant would cause slight physical injuries or mild illness in people, stock, and/or other desirable organisms, with no lasting effects.
- **None**—The plant would not affect the health of animals or people.
- **Impact question 6**. Could the plant have negative effects on environmental health?

The rating options are:

- **Yes**—Has major negative effects because it:

- **provides food/shelter to pests or pathogens**. For example, blackberry harbouring rabbits and grass weeds hosting wheat root diseases.
- **adversely changes the fire regime**. This includes changes to the normal frequency, intensity, and/or timing of fires.
- **adversely changes the nutrient levels**. For example, legumes can increase soil nitrogen. This may make native vegetation more prone to invasion by other plants, but would be beneficial in agriculture.
- **increases soil salinity**. If the leaves of the plant are high in salt, leaf decomposition may increase salinity at the soil surface.
- **adversely changes soil stability**. The plant increases soil erosion or silting of waterways.
- **adversely changes soil water table**. The plant substantially raises or lowers the soil water table compared to other plants present.

- **No**—Has minor or no negative effect on the factors above.

#### **Comments on impact questions**. 

Many GM crops under cultivation produce compounds that are toxic to certain insect pests. Classical risk assessment for these GM plants involves consideration of toxicity to any non-target organisms. Impact question 5 places more emphasis on whether there is harm to desirable organisms (e.g. stock, protected native animals or honey bees). However, some organisms may not be of great concern to society. For example, most countries approve the use of pesticides that kill a range of insects related to a target insect, even if these insects are not pests. Another example is that standard crop rotation practices in conventional agriculture drastically change the types and numbers of microorganisms in soil. Also note in relation to impact question 5 that although a plant may be toxic to humans or animals, if it is not palatable it may not actually be consumed and no harm from toxicity will eventuate.

Classical risk assessment of GM plants sometimes considers changes to soil function. Impact question 6 addresses changes to physicochemical characteristics such as nutrient levels, soil salinity, soil stability and soil water table levels. Effects on desirable soil organisms are covered by impact question 5. Note that GM plants may have an effect on soil stability by changing agricultural practices while a GM crop is being grown (e.g. no need for tillage). This effect may be adverse or beneficial.

### Invasiveness of plants

In chemical risk assessments, likelihood is termed exposure while the likelihood of infection with microorganisms is called infectivity. In weed risk assessment, the equivalent term is invasiveness (Standards Australia 2006). Invasiveness is the ability of a plant to spread and persist in the environment. The major components of invasiveness are establishment ability, reproductive ability, dispersal ability and potential distribution (Standards Australia 2006). Questions from the post-border weed risk assessment relating to the characteristics of plant invasiveness are shown below.

- **Invasiveness question 1**. What is the plant’s ability to establish amongst existing vegetation?

The rating options are:

- **Very high**—Seedlings readily establish within dense vegetation.
- **High**—Seedlings readily establish within more open vegetation.
- **Medium**—Seedlings mainly establish when there has been moderate disturbance to existing vegetation, which substantially reduces competition. This could include intensive grazing, mowing, raking, clearing of trees, temporary floods or summer droughts.
- **Low**—Seedlings mainly need bare ground to establish, including removal of stubble/leaf litter. This will occur after major disturbances such as cultivation, overgrazing, hot fires, grading, long-term floods or long droughts.

- **Invasiveness question 2**. What is the plant’s ability to survive to reproduction despite herbivory or pathogenesis?

The rating options are:

- **Very high**—Over 95 % of plants survive herbivory, insect pest and disease pressures. This level of survival could occur in exotic plants in the absence of their native pests and diseases.
- **High**—More than 50 % of plants survive herbivory, insect pest and disease pressures.
- **Medium**—Less than 50 % of plants survive herbivory, insect pest and disease pressures.
- **Low**—Less than 5 % of plants survive herbivory, insect pest and disease pressures.

- **Invasiveness question 3**. What is the plant’s tolerance to average weed management practices in the land use?

The rating options are:

- **Very high**—Over 95 % of plants survive commonly used weed management practices. For example, there may be no weed management practices in the relevant land use at the time when the plant grows.
- **High**—50 to 95 % of plants survive commonly used weed management.
- **Medium**—5 to 50 % of plants survive commonly used weed management.
- **Low**—Less than 5 % of plants survive commonly used weed management.

- **Invasiveness question 4**. What is the reproductive ability of the plant in the land use?

The rating options are:

- **Time to seeding**
**Short**—1 year
**Moderate**—2 to 3 years
**Long**—more than 3 years or never
- **Seed set**
**High**—more than 1,000 seeds per square metre
**Low**—less than 1,000 seeds per square metre
**None**—0 seeds
- **Vegetative production**
**Fast**—more than 10 new plants per year from a mature plant. In certain land uses, cultivation may increase vegetative reproduction by plant fragments.
**Slow**—less than 10 new plants per year from a mature plant
**None**—0 new plants

- **Invasiveness question 5**. How likely is long-distance dispersal of propagules (further than 100 m) by natural means?

The rating options are:

- **Common**—Dispersal occurs frequently via:

- **Birds**
- **Other wild animals**
- **Water**
- **Wind**
**Occasional**—Dispersal occurs sometimes by the vectors above.
**Unlikely**—Dispersal by natural means does not occur.

- **Invasiveness question 6**. How likely is long-distance dispersal (further than 100 m) by human means?

The rating options are:

- **Common**—Dispersal occurs frequently via:

- **Deliberate spread by people**. This includes planting for agriculture or gardens, or picking and discarding flowers.
- **Accidental spread by people and vehicles**
- **Contaminated produce**
- **Domesticated or farm animals**
**Occasional**—Dispersal occurs sometimes via the pathways above.
**Unlikely**—Dispersal by human means does not occur.

- **Invasiveness question 7**. What percentage of the land use is suitable for the plant?

The rating options are:

- **More than 80** **% of the land use is suitable for the plant considering climate, soil type and water availability.**
- **Between 60 and 80** **% of the land use.**
- **Between 40 and 60** **% of the land use.**
- **Between 20 and 40** **% of the land use.**
- **Between 10 and 20** **% of the land use.**
- **Between 5 and 10** **% of the land use.**
- **Between 1 and 5** **% of the land use.**
- **The plant is not suited to grow in any part of the land use.**

#### **Comments on invasiveness questions**.

A common trait of GM plants is resistance to certain insect pests. Invasiveness question 2 considers potential to survive insect pest pressures as one of several biotic stressors which impact on a plant’s ability to establish and survive. Insect pressure may or may not be the most important of these stressors. Similarly, some GM plants may have introduced resistance to particular pathogens.

Another common trait of GM plants is tolerance to certain herbicides. If these herbicides are part of the weed management regime in the relevant land use, the plant may have a higher invasiveness rating in regards to invasiveness question 3. However, risk assessors should also consider whether the GM plant could be easily controlled by other weed management practices if populations of the GMO became established.

Long-distance dispersal of GM plant propagules (seed and viable vegetative parts) is considered by invasiveness questions 5 and 6. Pollen dispersal is not covered by these questions, and is addressed in the section on gene transfer below.

Some GM plants are modified for increased water use efficiency, salt tolerance, or tolerance to other abiotic stressors, which may permit colonization of a larger proportion of a land use. This consideration is addressed by invasiveness question 7.

### Information requirements and outcomes

The questions posed in the PBWRA are outcome-focused. Therefore, each question can be addressed using a wide variety of data sources rather than prescribed test protocols. Weed risk assessment for GM plants can incorporate agronomic experience of the parent organism, molecular data, glasshouse studies and/or field observations.

In the PBWRA, impact and invasiveness questions are framed in terms that can be observed and measured. For example, in Invasiveness Question 4a, time to seeding is categorised (‘short’, ‘moderate’ or ‘long’) depending on the number of years between planting and seed production. The quantitative categories maintain the scientific integrity of the assessment and support evidence-based decision-making. In addition, the categories give guidance on where transitions between different levels of concern occur. For example, if a GM plant sets seeds slightly faster than the non-GM parent, but both plants fall in the same rating category (e.g. ‘short’ for annual plants), the change is not likely to affect the weed risk. On the other hand, if a biennial parent plant is genetically modified to become an annual plant (moving from ‘moderate’ to ‘short’ time to seeding), the altered trait in the GM plant may warrant further scrutiny in the risk assessment process.

By comparing the PBWRA of a GM plant to its non-GM parent, risk assessors can produce a list of the impact or invasiveness characteristics where the GM plant has a rating of higher concern than its unmodified parent species. This can provide a foundation for preparing risk scenarios based on the specific characteristics of the GM plant rather than generic risk scenarios.

### Gene transfer

Gene transfer includes movement of genes to plants of the same species by pollen flow, or to sexually compatible plants of different species by pollen flow, or to other organisms by horizontal gene transfer (Keese 2008).

Transfer of introduced genetic material from GM plants to other plants of the same species generally produces plants with the same characteristics as the GM plants, so does not require a separate PBWRA.

Transfer of introduced genetic material to sexually compatible plants of other species (or other subspecies with different traits) may be possible, if there is alignment of factors such as co-location, flowering times, availability of pollinators and if hybrids are viable. One simple way to apply the PBWRA to gene flow is to conduct an assessment for potential hybrids of a GM plant with recipient species. With this approach, where sexually compatible species may be able to acquire the genetic modification, the PBWRA should be completed for

- the non-GM parent species (baseline assessment)
- the GM plant (comparative assessment)
- any relevant sexually compatible species (baseline assessment) and
- potential hybrids of the GM plant with the sexually compatible species (comparative assessment).

Risk assessment of gene transfer to organisms via horizontal gene transfer cannot be assessed using a weed risk assessment approach. Instead, other approaches are required (Keese 2008).

### Unintended effects

In addition to the desired trait, genetic modification may give rise to unintended effects. The unintended changes may result in traits that are beneficial, adverse or neutral. Unintended effects leading to undesirable agronomic traits are usually deliberately excluded during the plant breeding process, and other unintended effects may be lost during back-crossing.

Weed risk assessment requires information about relevant characteristics of the GM plant. These may include changes due to intended and unintended effects. However, changes that have no or negligible effect on any of the PBWRA questions need not be explored. The PBWRA therefore provides guidance on the data requirements, for both intended and unintended traits, which are considered relevant for environmental risk assessment of a GM plant.

### Uncertainty

Environmental protection agencies apply the PBWRA to a wide range of plant species. Even for plant species with limited characterization, leading to a level of uncertainty, the PBWRA has been demonstrated to produce robust evaluations for regulatory decision-making (e.g. Government of South Australia 2013; New South Wales Department of Primary Industries 2013).

All GM plants to date are derived from cultivated plants, which are typically well characterized. Most of the characteristics of the parent species will be retained in the GM plant. Almost all of the questions in PBWRA of a GM plant can be readily answered from knowledge of the parent plant and of the intentional modification. Although there could be some uncertainty due to incomplete data about the introduced trait or possible unintended effects, the uncertainty is likely to be limited to one or a few questions. In most cases the uncertainty would not be of sufficient magnitude to potentially change the rating of a PBWRA characteristic of the GM plant compared to its parent. If the uncertainty is large enough that there could potentially be a negative change to the rating of a PBWRA characteristic, this characteristic should be incorporated into risk scenarios in the same way as if there was a known negative change to rating.

## Case study for applying PBWRA to a GM plant

Table 2 presents a case study demonstrating one potential approach for adapting PBWRA to a risk assessment of a hypothetical GM plant. The example is a proposed commercial release in Australia of GM cotton (*Gossypium hirsutum*) modified for insect resistance and tolerance to a herbicide. No known unintended traits are present.

**Table 2:** PBWRA of a hypothetical insect resistant and herbicide tolerant GM cotton, and relevant comparators

Land use	Plant	Rating
Impact question: could the plant affect the health of animals and/or people?		
Nature reserve	Non-GM cotton	**Low**. Cotton contains compounds that may be toxic if ingested in large quantities. However, most native animals find it unpalatable
	GM cotton	**Medium?** In addition to the above, GM cotton may kill insects that consume it due to toxicity from the insect resistance gene. There is uncertainty about whether any of these insects are desirable for nature conservation in a nature reserve land use
Irrigated/dryland agriculture	Non-GM cotton	**Low.** Cotton contains compounds that may be toxic if ingested in large quantities. However, cotton products are treated to remove toxins before human consumption, and stock are only fed cotton in safe quantities. Cotton is usually treated with pesticides to kill insects. In an agricultural setting these insects are not considered desirable
	GM cotton	**Low**. Similar to non-GM cotton. The pesticide compounds expressed in GM cotton would not kill a larger range of insects than standard pesticides (chemical or organic) applied to cotton
Invasiveness question: what is the plant’s ability to survive to reproduction despite herbivory or pathogenesis?		
Nature reserve	Non-GM cotton	**Low**. Cotton seedlings and young plants are susceptible to insect herbivory as well as disease and pathogens
	GM cotton	**Medium?** Although the statement above applies to GM cotton, the GM insect resistance trait potentially reduces herbivory of seedlings and young plants by some insects
Irrigated/dryland agriculture	Non-GM cotton	**Low**. Cotton seedlings and young plants are susceptible to insect herbivory as well as disease and pathogens. Agricultural areas may be treated with chemicals such as pesticides or fungicides to reduce pest or pathogen pressure
	GM cotton	**Medium?** Although the statements above apply to GM cotton, the GM insect resistance trait potentially reduces herbivory of seedlings and young plants by some insects
Invasiveness question: what is the plant’s tolerance to average weed management practices in the land use?		
Nature reserve	Non-GM cotton	**High**. In some nature conservation areas there are weed management practices, but these do not specifically target cotton. In other nature conservation areas no weed management is conducted
	GM cotton	**High?** As above. If weed management in nature conservation areas involves broad use of the specific herbicide that the GM cotton is tolerant to, the survival of GM cotton could potentially be increased
Irrigated/dryland agriculture	Non-GM cotton	**Low**. Cotton volunteers are typically controlled by mechanical methods such as mulching and root cutting and/or the application of appropriate herbicides
	GM cotton	**Medium?** Although GM cotton tolerates a certain herbicide, farmers who have planted GM cotton are unlikely to attempt to use this herbicide to control cotton volunteers in a subsequent crop. If there has been inadvertent gene flow from a GM cotton crop to another cotton crop, the volunteers from the other cotton crop could potentially survive standard herbicide application

Only impact or invasiveness questions where the GM cotton differs from its parent species are listed

This PBWRA methodology is used at an early stage in risk identification. It compares the properties of the GM cotton with the non-GM parent cotton in two relevant types of land use (receiving environments). These are dryland/irrigated agriculture where cotton is normally grown and nature reserves close to commercial growing sites. It is expected that the introduced genes are capable of gene transfer to commercial crops of Pima (Egyptian) cotton (*G. barbadense*), which is fully sexually compatible with *G. hirsutum* and overlaps in cultivation areas and flowering time. However, for all questions addressed in Table 2, the weediness characteristics of *G. barbadense* are very similar to those of *G. hirsutum*, so the two species are grouped as cotton. No other species in the Australian environment are sexually compatible with *G.* *hirsutum*.

**Fig. 1:**
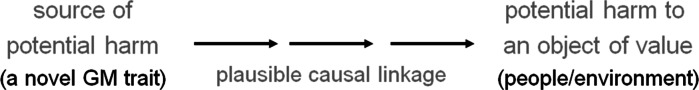
Components of a risk scenario

Five characteristics of possible concern emerge from the PBWRA case study comparing GM cotton to a baseline of non-GM cotton (Table 2). For some of these characteristics, the genetic modification is expected to cause a negative change to the rating; for others there is uncertainty about whether there could be a negative change (indicated by a question mark). Examples of risk scenarios (Fig. 1) that lead from the characteristics of the GM cotton to potential harm to people or the environment in a particular land use are listed in Table 3.

**Table 3:**
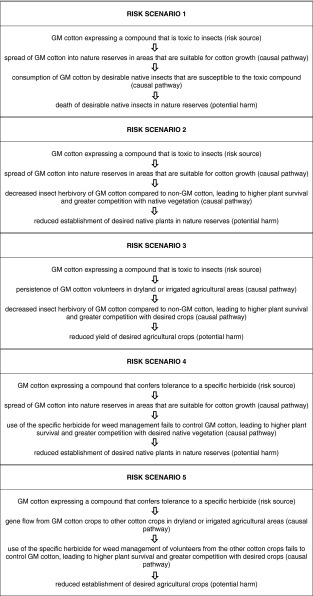
List of example risk scenarios postulated for insect resistant and herbicide tolerant GM cotton

### Risk scenarios derived from PBWRA

After postulation of risk scenarios, the next step in risk assessment would be to evaluate both the plausibility of the causal pathway and the severity of the potential harm for each risk scenario.

## Conclusions

The case study above illustrates the applicability of the PBWRA to risk assessment of GM plants. The PBWRA questions are formulated based on extensive experience from weeds, which ensures that known potential environmental risks from plants, including GM plants, will be considered. This framework provides a rational way to identify essential information for conducting environmental risk assessment. In addition, the PBWRA ratings provide guidance on when a difference between a GM plant and its parent is likely to be significant. This assists risk assessors to focus only on risk scenarios where the genetic modification could plausibly lead to harm.

The PBWRA provides a coherent framework that specifies the key characteristics of plants that affect invasiveness and the types of impacts that are considered adverse. It delivers a robust, validated approach for risk assessment of any type of potentially problematic plant, including GM plants. Using the PBWRA would bring risk assessment of GM plants in line with the terminology and approaches used in assessing similar risks for other plants.
